# Comparison of Large Language Model Performance on the United Kingdom Neurology Specialty Certificate Examination

**DOI:** 10.7759/cureus.112413

**Published:** 2026-07-10

**Authors:** Josiah Cho, Arvindh Sekaran

**Affiliations:** 1 Medicine, Addenbrooke's Hospital, Cambridge University Hospitals NHS Foundation Trust, Cambridge, GBR

**Keywords:** ai, large language model, medical education, neurology, specialty certificate examination

## Abstract

Background

Large language models (LLMs) are increasingly used in medical education; however, a gap remains in the literature comparing the performance of different LLMs on neurology-specific higher-specialty training assessments. This study aimed to compare the performance of three leading LLMs, including ChatGPT (OpenAI, San Francisco, CA, USA), Claude (Anthropic, San Francisco, CA, USA), and Gemini (Google LLC, Mountain View, CA, USA), on UK Neurology Specialty Certificate Examination (SCE) sample questions.

Materials and methods

This comparative cross-sectional performance evaluation used 83 official UK Neurology SCE single-best-answer (SBA) questions obtained from the Membership of the Royal Colleges of Physicians of the UK (MRCP (UK)). Explicit permission was obtained from MRCP (UK) for research and publication. Evaluated models included ChatGPT (version 5.5, thinking mode), Claude (Opus 4.6), and Gemini (Pro, thinking mode). Each LLM was presented with an identically formatted question set and identical prompts, instructing it to select the SBA and provide a brief explanation limited to 200 words. Each question was entered into a new chat session, so responses were generated independently of previous items. Official answers were used as the reference standard. Responses were coded as correct or incorrect, with binary answer accuracy as the primary outcome. Mixed-effects logistic regression compared the probability of a correct answer between LLMs, with question ID as a random intercept to account for question-level variation. Pairwise comparisons were adjusted using Holm correction to account for multiple testing. Confirmatory paired analyses were performed using Cochran’s Q test and exact McNemar tests.

Results

ChatGPT answered 78 of 83 questions correctly, corresponding to an accuracy of 94.0% (95% Wilson CI 86.7-97.4%). Gemini also answered 78 of 83 questions correctly, with an accuracy of 94.0% (95% Wilson CI 86.7-97.4%), while Claude answered 66 of 83 questions correctly, corresponding to a lower accuracy of 79.5% (95% Wilson CI 69.6-86.8%). The mixed-effects model demonstrated a significant association between LLM identity and answer accuracy. Confirmatory paired analyses supported these findings. Cochran’s Q test indicated a significant overall difference in accuracy among models (Q = 16.94, df = 2, p = 0.00021). Exact McNemar tests confirmed that ChatGPT and Gemini both significantly outperformed Claude, with no significant difference between them.

Conclusions

ChatGPT and Gemini demonstrated identical accuracy on official UK Neurology SCE sample questions, while Claude achieved notably lower accuracy on this dataset. Together, these findings suggest LLMs may perform well on selected specialist neurology training assessment questions and favor their use as a supplementary learning tool. However, these findings are based on a relatively small sample of questions and, in isolation, do not translate into real-world clinical competence or reasoning ability. Further studies should evaluate the quality of LLM clinical reasoning through predefined mark schemes and specialty-specific expert review.

## Introduction

Over the last decade, advances in artificial intelligence (AI) have led to a global increase in its use. In particular, the use of large language models (LLMs) has scaled rapidly, with LLMs such as ChatGPT alone having reached over 900 million weekly active users as of early 2026 [1]. The growing incorporation of AI into education has also introduced valuable new opportunities for personalized learning, feedback, and instructional support. Thus, numerous studies have explored the use of AI to supplement traditional methods in medical education for teaching, assessment, clinical reasoning, and even for facilitating admissions processes [2]. For example, AI performance has been evaluated in simulated patient interactions, intelligent tutoring systems, and the generation and assessment of multiple-choice questions [2].

In specialist medicine, AI has so far shown the greatest potential in imaging-dominated specialties, including radiology, pathology, and ophthalmology. For example, a 2022 systematic review of over 500 radiology AI articles suggested promising diagnostic capabilities of AI for tumor classification using imaging [3]. Similarly, AI has performed well in whole-slide pathology-based diagnosis, including renal and prostate cancer [4], as well as in detecting pathological myopia on fundus imaging [5]. In neurology, AI has shown proficiency in diagnostic neuroimaging, particularly in stroke medicine. A 2024 meta-analysis of 33 studies found that AI accurately detected ischemic stroke on magnetic resonance imaging, with sensitivity and specificity of 93% [6]. Taken together, the versatility and performance of AI so far highlight its emerging potential to complement learning in medical education and diagnostic settings.

However, while AI has demonstrated promising opportunities in education and across multiple medical specialties, its translation into routine clinical practice remains limited by several barriers. Firstly, patient confidentiality is a recurring concern in the literature. AI algorithms require access to large datasets for learning, which poses a potential risk to patient privacy; explicit consent should be obtained to use their information [7]. Liability issues also remain under debate regarding where the ultimate responsibility lies for the consequences of clinical decisions influenced by AI [7]. Additionally, obstacles at the clinician level, including the training and education required to enable practitioners to use AI in healthcare competently, pose barriers to the translation of AI diagnostics into routine clinical practice [7]. Addressing confidentiality concerns and determining the appropriate role of AI in healthcare remain ongoing challenges for its implementation into real-world clinical practice.

LLM performance has been evaluated across a range of UK postgraduate medical examinations, including the Membership of the Royal Colleges of Physicians of the UK (MRCP UK) Part 1 [8], Multi-Specialty Recruitment Assessment [9], and Membership of the Royal College of Surgeons [10] examinations, attaining scores above the historical pass mark threshold each time. The current literature, however, is more limited in its coverage of LLM proficiency in specialty-specific higher-specialty training assessments and in comparative studies evaluating the capabilities of different LLMs. ChatGPT's performance on questions from the UK Neurology Specialty Certificate Examination (SCE) [11] and UK Dermatology SCE [12] has been assessed, with successful scores each time. By contrast, Fan and colleagues conducted a comparative evaluation of seven LLMs on the UK Diabetes and Endocrinology SCE, revealing inadequate performance by six of the seven LLMs tested [13]. This was likely attributed to the relative weakness of LLMs in multi-step clinical reasoning and decision-making compared with their strengths in factual recall. However, a distinct gap remains in the literature regarding the comparative performance of LLMs in neurology-specific higher-specialty training assessments.

The UK Neurology SCE is a standardized, computer-based postgraduate assessment comprising 200 single-best-answer (SBA) questions designed to evaluate the competency of physicians in neurology specialty training prior to consultant qualification. This study aimed to compare the performance of three leading LLMs in the UK Neurology SCE, with a view to assessing the potential of LLMs as an educational tool in neurology specialist medical training.

## Materials and methods

Study design and dataset

**Figure 1:**
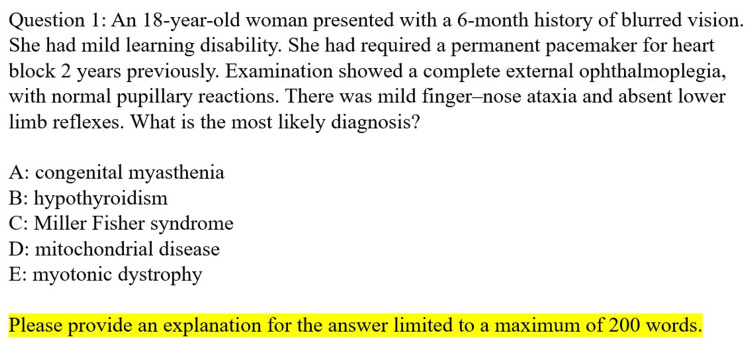
Example of a question and standardized prompt (highlighted in yellow) used for LLM assessment LLM: large language model

In this comparative cross-sectional performance evaluation study, we assessed the accuracy of three LLMs in a set of 83 UK Neurology SCE SBAs. Evaluated models included ChatGPT (version 5.5, thinking mode; OpenAI, San Francisco, CA, USA), Claude (Opus 4.6; Anthropic, San Francisco, CA, USA), and Gemini (Pro, thinking mode; Google LLC, Mountain View, CA, USA). All questions were obtained from the official UK Neurology SCE sample questions published by MRCP (UK). Explicit permission was obtained from MRCP (UK) to use this resource for this study and for publication of the results. To ensure consistency across models, all LLMs were presented with an identically formatted, standardized version of the question set and given identical prompts to select the SBA and provide a brief explanation limited to 200 words (Figure 1).

For all models, each question was entered into a new chat to ensure responses were generated independently of previous items. All prompts were placed and responses collected on a single day (April 17, 2026). The correct answer provided for each question from the official sample paper was used as the reference standard, and the unit of analysis was the model-question pair. Binary coding was used for each model's responses: 1 (correct), 0 (incorrect). The primary outcome was defined as binary answer accuracy. Text explanations given by each model were recorded but not included in the analysis. Repeated runs were not performed because the study aimed to assess each LLM's single-attempt performance, similar to an exam setting.

Analysis

Primary Statistical Analysis

All data analyses were performed in R (R Foundation for Statistical Computing, Vienna, Austria). A mixed-effects logistic regression model was used to compare the probability of a correct answer across LLMs. Question ID was included as a random intercept, as the questions answered by each model were identical, and individual questions may have varied in difficulty. Model effects were reported as odds ratios with 95% confidence intervals (CI). We first evaluated whether there was any overall evidence that accuracy differed among the three LLMs before examining pairwise comparisons between individual models. The full mixed-effects logistic regression model with LLM identity (ChatGPT, Claude, or Gemini) was compared with a null model that included only the question ID random intercept. Using marginal contrasts, the mixed-effects model was then applied to estimate pairwise comparisons between LLMs. The Holm correction was used to adjust p-values to account for multiple testing. Individual model accuracy was also summarized as the number and percentage of correctly answered questions, with 95% Wilson CIs.

Confirmatory Paired Analyses

Paired binary tests were conducted for confirmatory analyses as each LLM answered an identical set of questions. Cochran’s Q test was employed to assess whether there was an overall difference in accuracy between the three models. Exact McNemar tests were then used for pairwise comparisons between models, with Holm correction used to adjust for multiple comparisons. All statistical tests were two-sided, with p < 0.05 defined as statistically significant.

## Results

**Figure 2:**
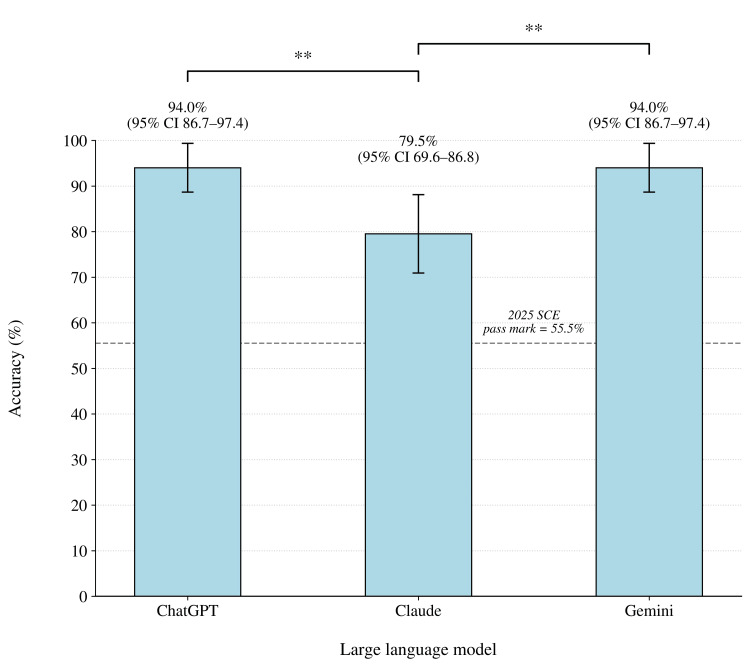
Accuracy by LLM CI: confidence interval, SCE: Specialty Certificate Examination, LLM: large language model

**Figure 3:**
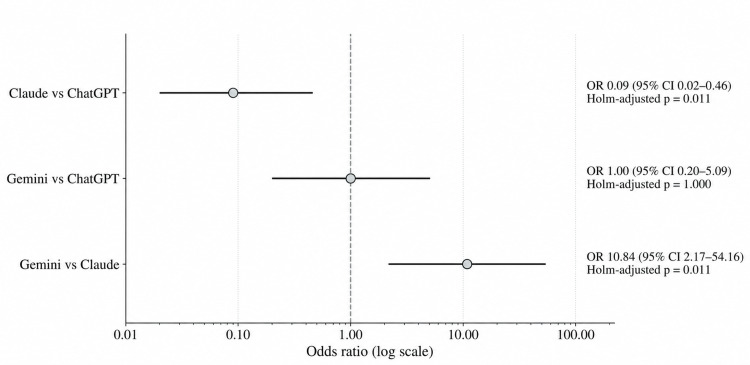
Mixed-effects logistic regression pairwise contrasts OR: odds ratio, CI: confidence interval

A total of 83 UK Neurology SCE sample questions were included. ChatGPT answered 78 of 83 questions correctly, corresponding to an accuracy of 94.0% (95% Wilson CI 86.7-97.4%) (Figure 2). Gemini also answered 78 of 83 questions correctly, with the same accuracy of 94.0% (95% Wilson CI 86.7-97.4%). Despite achieving identical overall accuracy, ChatGPT and Gemini produced discordant outcomes on four questions, with one model answering correctly and the other incorrectly (Table 1). Claude answered 66 of 83 questions correctly, corresponding to an accuracy of 79.5% (95% Wilson CI 69.6-86.8%). Primary analysis using the mixed-effects logistic regression model revealed that LLM identity was significantly associated with the probability of a correct answer after accounting for question-level clustering and variation in question difficulty. A likelihood-ratio comparison of the mixed-effects model that includes LLM identity with the null random-intercept model revealed a statistically significant difference in answer accuracy among the three LLMs after accounting for question-level variation. Compared with ChatGPT, Claude had significantly lower odds of answering correctly (OR 0.09, 95% CI 0.02-0.46, p = 0.0037; Holm-adjusted p = 0.011) (Figure 3). Gemini did not differ significantly from ChatGPT (OR 1.00, 95% CI 0.20-5.09, p = 1.000). Gemini had significantly higher odds of answering correctly compared with Claude (OR 10.84, 95% CI 2.17-54.16, p = 0.0037; Holm-adjusted p = 0.011). The random-intercept standard deviation for the question was approximately 2.66, suggesting there was substantial variation in question-level difficulty.

**Table 1:** Questions with discordant accuracy between ChatGPT and Gemini Discordant outcomes were defined as questions in which one model answered correctly and the other incorrectly.

Question	Correct answer	ChatGPT answer	Gemini answer
Question 38: A 24-year-old woman presented with a 2-month history of morning headaches. On examination, she had bilateral papilloedema. What is the most characteristic visual symptom associated with this sign? (A: blackening of whole of vision on standing, B: flashing white dots when eyes are closed, C: general blurring of vision, D: greying of vision on bending forward, E: tunnel vision with coughing)	D	A	D
Question 43: A 77-year-old man presented with abnormal movements, first noted after a knee replacement 3 years previously. His memory had deteriorated, his coordination had become impaired, and his speech had become unintelligible. He was the oldest of three brothers. His father had died at the age of 64, and his mother at 73. He had no children. On examination, there was generalized chorea, although mainly in his head and neck, with pouting mouth movements. His speech was slurred, dysphonic and 'explosive'. The limb reflexes were brisk, and he was ataxic. What is the most likely diagnosis? (A: chorea acanthocytosis, B: dentatorubral-pallidoluysian atrophy, C: Huntington’s disease, D: neuroferritinopathy, E: spinocerebellar ataxia type 17)	C	C	E
Question 45: A 54-year-old woman gave a 1-week history of recurrent dizziness and unsteadiness. Her symptoms were worse when she first sat up in the morning. They had begun after a road traffic collision. Her medical history included type 2 diabetes mellitus, hypercholesterolemia and hypertension. She smoked 30 cigarettes per day. On examination, she appeared anxious. There were no focal neurological signs. A CT scan of the head was normal. What is the most likely diagnosis? (A: benign paroxysmal positional vertigo, B: cervicogenic dizziness, C: perilymph fistula, D: persistent posturo-perceptual dizziness, E: vertebral artery dissection)	A	A	E
Question 63: A 24-year-old soldier was involved in an ambush during which the car he was traveling in was blown up by a land mine. He sustained minor limb and facial injuries, but the other two occupants of the car were killed. Two weeks after the incident, he complained of intermittent headaches, nightmares, poor concentration and anxiety. He expressed guilt at being the only person to survive the explosion and expressed a wish to leave the Army. On examination, he was distressed and tearful. What is the most likely diagnosis? (A: acute depressive episode, B: adjustment disorder, C: factitious disorder, D: panic disorder, E: post-traumatic stress disorder)	B	E	B

Results from the paired confirmatory analyses supported those of the primary model (Table 2). Cochran’s Q test indicated a significant overall difference in accuracy among the three models (Q = 16.94, df = 2, p = 0.00021). In exact McNemar pairwise comparisons with Holm correction, ChatGPT significantly outperformed Claude (adjusted p = 0.0055). Similarly, Gemini also performed significantly better than Claude (adjusted p = 0.0084). There was no significant difference between ChatGPT and Gemini (adjusted p = 1.000).

**Table 2:** Confirmatory paired analyses All p-values are two-sided. Statistical significance for Cochran's Q test was defined as p < 0.05, indicated by an asterisk (*). Statistical significance for exact McNemar tests was defined as Holm-adjusted p < 0.05, indicated by an asterisk (*). df: degrees of freedom

Analysis	Comparison	Statistic/discordant pairs	p-value	Holm-adjusted p-value
Cochran’s Q	Overall	Q = 16.94; df = 2	0.00021*	-
Exact McNemar	ChatGPT vs Claude	13 vs 1	0.0018	0.0055*
Exact McNemar	ChatGPT vs Gemini	2 vs 2	1.00	1.00
Exact McNemar	Claude vs Gemini	2 vs 14	0.0042	0.0084*

## Discussion

These results demonstrated the superior performance of ChatGPT and Gemini on 83 UK Neurology SCE sample questions, with each scoring 94.0% correctly, compared with Claude, which scored 79.5%. Mixed-effects logistic regression revealed that LLM identity was significantly associated with answer accuracy after adjusting for variation in question-level difficulty, consistent with confirmatory paired analyses. Pairwise comparisons demonstrated that ChatGPT and Gemini significantly outperformed Claude, while there was no significant difference between them.

Together, these findings suggest that LLMs may perform well on selected specialist neurology training assessment questions. As a useful reference, the pass mark for the 2025 UK Neurology SCE was 55.5% [14]. However, direct comparison between performance on a relatively small sample of questions and the official examination should be interpreted with caution. The findings from this study potentially support the growing use of LLMs in education, including in higher-specialty training assessments. LLMs may serve as a useful learning tool to supplement revision by providing explanations and individualized feedback [15]. However, further studies evaluating the quality of explanations would be required to support this. Additionally, the use of LLMs in clinical practice should be approached with caution, as SBA performance alone will not necessarily translate into clinical competence in real-life settings, where decision-making is often multifactorial and requires overall consideration of history, examination, patient autonomy, ethical issues, risk-benefit analyses, and best interests [16,17]. Therefore, LLMs should be used as a supplementary educational tool rather than a replacement for clinical practice.

There are several limitations to this study. Firstly, the dataset was restricted to 83 sample questions, limiting statistical power and the ability to detect small differences between high-performing models. Larger datasets would also enable more reliable analysis of performance by question type and topic. Secondly, as the question set was derived from an official sample paper, rather than a full examination, the selection of questions may not be fully representative of the breadth or difficulty of the UK Neurology SCE. Thirdly, only final answer accuracy was evaluated in this study, and not the quality of explanation provided by each AI engine. It is possible a correct final answer was provided via flawed reasoning, as found in similar studies [18]. Conversely, an incorrect final answer may have been provided with a plausible clinical explanation. Finally, although new chat sessions were used to minimize contamination from previous items, it was not possible to assess whether similar content had been used in model training. This is a potentially important limitation, as exposure to publicly available sample questions during training could have contributed to performance independent of true clinical reasoning ability. Further studies should compare LLM performance on larger neurology question sets, in which newly generated questions are used to prevent training-data contamination. The quality of explanations provided by each model should also be evaluated through blinded expert reviews under predefined mark schemes.

## Conclusions

In this comparative evaluation of three LLMs across 83 UK Neurology SCE SBAs, ChatGPT and Gemini achieved identical accuracy, and both significantly outperformed Claude. Mixed-effects logistic regression, adjusted for question-level variation, showed a significant association between LLM identity and accuracy, with confirmatory paired analyses supporting these findings. These results suggest LLMs may have value as a supplementary learning tool for specialist neurology training assessment. However, these results are based on a small dataset of questions and, in isolation, do not necessarily translate to real-world clinical competence or reasoning ability in neurology. Further studies should evaluate the quality of clinical reasoning provided by LLMs through predefined mark schemes and neurology-specific expert review.
